# Impact of prehabilitation on quality of life and mental health in patients awaiting cytoreductive surgery for ovarian cancer or suspected adnexal tumors

**DOI:** 10.1186/s12905-026-04490-3

**Published:** 2026-05-25

**Authors:** Marcin Adam Zębalski, Paula Szostek, Jakub Szpiech, Aleksandra Krzywon, Krzysztof Nowosielski

**Affiliations:** 1 Department of Gynecology, Obstetrics and Gynecological Oncology, University Clinical Center of the Medical University of Silesia, Katowice, 40-752 Poland; 2 Department of Biostatistics and Bioinformatics, Maria Sklodowska-Curie National Research Institute of Oncology, Gliwice Branch, Gliwice, 44-102 Poland; 3 Department of Applied Informatics, Silesian University of Technology, Gliwice, 44- 100 Poland

**Keywords:** Prehabilitation, Ovarian cancer, Quality of life, Depression, Anxiety

## Abstract

**Background:**

Preoperative psychological distress and impaired quality of life are common in patients awaiting surgery for ovarian cancer or adnexal masses. We aimed to assess whether a multimodal prehabilitation program including physical exercise, nutritional support, and psychological counseling could improve quality of life and reduce anxiety and depression prior to surgery.

**Methods:**

In this prospective single-centre study, 145 patients were enrolled from May 2021 to May 2024 at a gynecologic oncology centre in Katowice, Poland. Baseline and post-intervention assessments included the European Organisation for Research and Treatment of Cancer Quality of Life Questionnaire (EORTC QLQ-C30) and the Hospital Anxiety and Depression Scale (HADS). The prehabilitation program lasted on average 21.1 ± 16.7 days and combined physical exercise, nutritional support, and psychological counseling.

**Results:**

After completion of prehabilitation, global health status/quality of life scores improved modestly but significantly (mean change  ~2.5 points; *p* = 0.045). Mean HADS scores declined for anxiety (*p* = 0.003) and for depression (*p* = 0.028), indicating reductions in psychological distress.

**Conclusions:**

Participation in a structured prehabilitation program was associated with improvements in overall quality of life and reductions in anxiety and depression in women awaiting gynecologic oncologic surgery, although the magnitude of these changes was modest. Notably, these improvements were observed despite the substantial psychological burden typically experienced during the preoperative period. These findings support incorporation of prehabilitation into standard preoperative care to better prepare patients both physically and mentally.

## Introduction

In recent years, the incidence of malignant tumors, including gynecologic cancers, has continued to rise, while both overall survival and disease-free survival have also improved in many cancer types. As a result, an increasing number of patients are living longer with cancer, often for extended periods. Cancer and its treatment can affect multiple aspects of functioning, including physical, psychological, and social well-being.

The interval between an initial cancer diagnosis and definitive surgery represents a particularly stressful period. The mere suspicion of malignancy can worsen quality of life and heighten fears about the future. The interval between diagnosis and treatment has recently been the subject of increasing scientific interest. A recent systematic review by Zouzoulas et al. demonstrated that prolonged delays in initiating treatment for ovarian and endometrial cancers may negatively influence survival outcomes and are also associated with increased psychological distress and deterioration in quality of life [1]. These findings highlight the clinical and psychological importance of the preoperative period and emphasize the need for interventions that may mitigate its negative impact on patients. Ovarian cancer is an especially aggressive disease that is typically diagnosed at an advanced stage. Its poor prognosis and rapid progression frequently lead to increased stress, anxiety, and depressive symptoms, negatively affecting quality of life [2]. Numerous studies have shown that cancer diagnosis and upcoming surgery are associated with poorer quality of life and increased anxiety and depression [3–9].

Prehabilitation refers to a set of preoperative interventions designed to enhance a patient’s functional capacity and thereby reduce postoperative complications [10–12]. Although most published reports focus on physical exercise and nutritional optimization, psychological preparation is an equally important pillar. To date, however, the mental health component of prehabilitation has been less extensively investigated.

While many studies suggest that prehabilitation improves postoperative outcomes, there is no consensus regarding the optimal program or its effectiveness in different patient populations. Implementation may be hindered by factors such as frailty, disease progression, or psychological distress related to a cancer diagnosis. We hypothesize that depressive symptoms and heightened anxiety can significantly compromise engagement in prehabilitation.

This study provides a prospective evaluation of the psychological impact of multimodal prehabilitation by assessing changes in quality of life, anxiety, and depression in patients awaiting gynecologic oncologic surgery.

The present study aimed to evaluate changes in quality of life and the severity of anxiety and depression among patients awaiting surgery for suspected gynecologic malignancy. We hypothesized that participation in a multimodal prehabilitation program would improve quality of life and reduce preoperative anxiety and depression.

## Materials and methods

### Patients

The study included patients referred for surgical treatment of ovarian cancer or suspicious adnexal lesions between 5 May 2021 and 23 May 2024 at the Department of Gynecology, Obstetrics and Oncologic Gynecology, Katowice, Poland. All patients scheduled for surgery were offered participation in a prehabilitation program. Exclusion criteria included lack of consent to participate, severe neurological disorders preventing adherence to recommendations, and dementia.

### Prehabilitation program

The multimodal prehabilitation program was initiated during an introductory outpatient visit approximately two to four weeks before surgery. At baseline, patients underwent assessment of physical fitness (including the 6-Minute Walk Test), nutritional status, and symptoms of anxiety and depression. Laboratory tests (complete blood count, liver and kidney function, thyroid profile, vitamin D, total protein, and albumin) were performed, and any abnormalities were corrected or referred for specialist management.

The program consisted of five key components:

- Physical activity: Patients received individualized instructions for home-based resistance and aerobic exercises aimed at improving core and overall muscle strength as well as cardiorespiratory fitness. Exercises were to be performed daily or every other day, with gradual intensity progression and self-monitoring.
- Nutrition and protein supplementation: Participants were advised to follow a high-protein diet (1.5–2.0 g/kg/day), limit simple carbohydrates, increase vegetable intake, and incorporate protein supplements enriched with immune-supporting nutrients (e.g., omega-3 fatty acids, beta-glucan) when appropriate.
- Lifestyle modification: Smoking cessation and alcohol reduction were strongly encouraged, and supportive counseling was provided when necessary.
- Psychological support: Patients scoring ≥ 8 on the Hospital Anxiety and Depression Scale (HADS) or requesting assistance were referred for counseling to address anxiety and depressive symptoms during the preoperative period.
- Medical optimization: Identified abnormalities (e.g., anemia, vitamin D deficiency, thyroid disorders) were treated prior to surgery.

All patients received printed educational materials and access to online instructional videos to promote adherence. The program was designed to complement the standard Enhanced Recovery After Surgery (ERAS) protocol implemented for all participants. Adherence to the recommended prehabilitation activities was monitored through patient self-reports, follow-up telephone conversations, and exercise diaries documenting the frequency of performed exercises. Patients were asked to bring their exercise diaries on the day of hospital admission.

The prehabilitation program followed our previously published protocol [13], with essential details provided herein.

### Assessment of quality of life, anxiety, and depression

During the introductory visit, each patient completed the EORTC QLQ-C30 questionnaire and the Hospital Anxiety and Depression Scale (HADS).

The European Organisation for Research and Treatment of Cancer Quality of Life Questionnaire (EORTC QLQ-C30) objectively assesses quality of life in functional and symptomatic domains among oncological patients. It includes five functional domains (physical, role, cognitive, emotional, and social), three symptom domains (fatigue, pain, and nausea/vomiting), a global health status/quality of life (GHS/QoL) domain, and five single-item domains (dyspnea, insomnia, appetite loss, constipation, diarrhea, and financial difficulties).

Higher scores in the functional and global health domains indicate better quality of life, while higher symptom scores reflect greater symptom burden and poorer quality of life [14].

The HADS is a self-report questionnaire with two subscales designed to identify and quantify anxiety and depression, particularly in oncological populations [15]. Patients scoring 8 or more points on either subscale were referred to a psychologist. Those with severe depressive symptoms unresponsive to psychological counseling were referred to a psychiatrist.

Reassessment of quality of life and psychological distress was performed on hospital admission, one day before surgery. Psychological care was also provided during the hospital stay.

### Statistical analysis

Categorical variables were summarized as frequencies and percentages, while continuous variables were expressed as medians with interquartile ranges (IQR, 25–75%). Differences between groups were evaluated using the Wilcoxon rank-sum test for continuous variables and Fisher’s exact test for categorical variables. Changes in parameters between baseline and pre-surgery assessments were analyzed using the one-sample Wilcoxon signed-rank test.

Correlations between variables were examined using Spearman’s correlation coefficients, classified as follows: 0.0 ≤ |r| < 0.1 (negligible), 0.1 ≤ |r| ≤ 0.39 (weak), 0.4 ≤ |r| ≤ 0.69 (moderate), 0.7 ≤ |r| ≤ 0.89 (strong), and 0.9 ≤ *r* ≤ 1.0 (very strong) [16]. A multivariable linear regression model was evaluated to determine the effects of age and disease stage on overall quality of life changes during the prehabilitation program. Statistical significance was defined as a two-sided p-value < 0.05. All analyses were performed in the R environment for statistical computing, version 4.0.1 (“See Things Now”), released on 6 June 2020 by the R Foundation for Statistical Computing, Vienna, Austria (http://www.r-project.org, accessed 29 February 2020).

The sample size was calculated for a paired samples t-test, reflecting the prospective design of the study (beginning and end of the prehabilitation program). The calculations assumed a significance level of α = 0.05 and a statistical power of 0.9. To detect a medium effect size (d = 0.5), which corresponds to a moderate clinically meaningful difference of 10 points on the Global Health Status scale (EORTC QLQ-C30), a minimum sample size of 44 patients was required [17].

## Results

### Patients

The study included 145 patients. Ovarian cancer was confirmed in 128 patients (88%), while in 17 patients the histopathological examination revealed benign adnexal lesions. The mean duration of the prehabilitation program was 21.1 days (SD 16.7). The general characteristic of the patients included in the study is presented in Table 1.

**Table 1:** General characteristics of the patients included in the study

Characteristic	Value (*n* total = 145)
Age (years), median [IQR]	63 (51, 69)
BMI (kg/m^2^), median [IQR]	26.4 (23.4, 29.8)
Menopause, (%)	103 (71)
Nulliparous, (%)	20 (13.8)
Tertiary education, (%)	52 (35.9)
Working patients, (%)	59 (40.7)
Physical activity^a^, (%)	38 (26.2)
ECOG 0, (%)	114 (78.6)
ECOG 1, (%)	26 (17.9)
Active smoker, (%)	30 (20.7)
Hypertension, (%)	73 (50.3)
Ischemic heart disease, (%)	21 (14.5)
Diabetes, (%)	20 (13.8)
Asthma/chronic obstructive pulmonary disease, (%)	5 (3.4)

*Abbreviations*: *BMI* Body Mass Index, *IQR* Interquartile range

^a^Regular engagement in ≥ 150 min of moderate-intensity physical activity per week qualified a patient as physically active

### Quality of life

Quality of life assessment based on the EORTC QLQC30 questionnaire was performed during the first visit and on the day of hospital admission. To better interpret the clinical relevance of the observed changes, we referred to the minimal clinically important difference (MCID) for the EORTC QLQ-C30. Previous studies indicate that a change of approximately 5–10 points in most domains of this questionnaire may be considered clinically meaningful [18]. We demonstrated an improvement in the overall quality of life during the prehabilitation program (*p* = 0.01). However, the observed improvement in global quality of life did not reach the minimal clinically important difference described for the EORTC QLQ-C30. Therefore, although the change was statistically significant, its clinical relevance may be modest. In the individual domains of quality of life, we demonstrated an improvement in global health status, physical functioning and emotional functioning (*p* < 0.05). We did not demonstrate any changes in role functioning, cognitive functioning and social functioning (*p* > 0.05). Among the variables from the symptom scales/items category, we found lower intensity of fatigue, nausea and vomiting, sleep problems, and loss of appetite. The prehabilitation program did not affect symptoms related to pain, dyspnoea, complaints related to constipation or diarrhea, or financial problems. The summary results of the patients’ quality of life based on the EORTC QLQ-C30 questionnaire before starting the prehabilitation program and after completing the prehabilitation program are presented in Table 2.

**Table 2:** Comparison of quality of life results during the prehabilitation program

Characteristic	Beginning mean (±SD) median (IQR)	End mean (±SD) median (IQR)	Difference mean (±SD) median (IQR)	*p*-value *
Global health status/QoL	54.6 (19.0) 50.0 (41.7, 66.7)	57.5 (19.4) 58.3 (50.0, 66.7)	2.5 (16.6) 0.0 (0.0, 8.3)	0.045 (0.20, small)
Physical functioning	80.2 (17.0) 86.7 (66.7, 93.3)	82.4 (15.9) 86.7 (73.3, 93.3)	2.2 (9.5) 0.0 (0.0, 6.7)	0.007 (0.23, small)
Role functioning	76.1 (25.1) 83.3 (66.7, 100.0)	78.5 (22.9) 83.3 (66.7, 100.0)	2.4 (20.1) 0.0 (0.0, 0.0)	0.19
Emotional functioning	64.9 (22.1) 66.7 (50.0, 83.3)	68.9 (19.2) 66.7 (58.3, 83.3)	3.5 (18.3) 0.0 (-8.3, 16.7)	0.03 (0.16, small)
Cognitive functioning	80.8 (20.7) 83.3 (66.7, 100.0)	82.7 (17.8) 83.3 (66.7, 100.0)	1.3 (16.7) 0.0 (0.0, 16.7)	0.34
Social functioning	76.6 (22.2) 83.3 (66.7, 100.0)	78.0 (21.6) 83.3 (66.7, 100.0)	0.8 (18.2) 0.0 (0.0, 0.0)	0.75
Fatigue	33.9 (22.2) 33.3 (22.2, 44.4)	30.2 (21.3) 33.3 (11.1, 44.4)	-3.7 (16.7) 0.0 (-11.1, 0.0)	0.02 (0.23, small)
Nausea and vomiting	7.6 (15.0) 0.0 (0.0, 16.7)	5.7 (12.4) 0.0 (0.0, 0.0)	-1.9 (11.6) 0.0 (0.0, 0.0)	0.04 (0.18, small)
Pain	27.1 (24.3) 33.3 (0.0, 33.3)	25.2 (22.8) 16.7 (0.0, 33.3)	-1.9 (18.8) 0.0 (0.0, 0.0)	0.21
Dyspnoea	14.4 (23.3) 0.0 (0.0, 33.3)	14.9 (24.7) 0.0 (0.0, 33.3)	0.5 (23.2) 0.0 (0.0, 0.0)	0.70
Insomnia	36.6 (27.4) 33.3 (0.0, 66.7)	30.7 (29.0) 33.3 (0.0, 33.3)	-5.9 (23.0) 0.0 (-33.3, 0.0)	0.003 (0.25, small)
Appetite loss	23.9 (27.7) 33.3 (0.0, 33.3)	18.7 (23.7) 0.0 (0.0, 33.3)	-5.2 (24.6) 0.0 (-33.3, 0.0)	0.02 (0.21, small)
Constipation	19.1 (27.1) 0.0 (0.0, 33.3)	18.4 (26.6) 0.0 (0.0, 33.3)	-0.7 (22.3) 0.0 (0.0, 0.0)	0.63
Diarrhoea	8.7 (16.2) 0.0 (0.0, 0.0)	7.1 (14.3) 0.0 (0.0, 0.0)	-1.7 (13.4) 0.0 (0.0, 0.0)	0.15
Financial difficulties	17.0 (23.1) 0.0 (0.0, 33.3)	15.8 (22.1) 0.0 (0.0, 33.3)	-1.4 (14.8) 0.0 (0.0, 0.0)	0.26
QLQTOTAL**	77.5 (14.2) 79.6 (70.8, 87.6)	80.0 (13.4) 82.7 (71.3, 90.4)	1.9 (12.0) 0.2 (-2.6, 8.2)	0.01 (0.20, small)

*Abbreviations*: *SD* Standard deviation, *IQR* Interquartile range, *QoL* Quality of life

^*^Effect size reported only for *p* < 0.05 (interpretation)

^**^EORTC QLQ-C30 scores range from 0 to 100. Higher scores in functional and global health domains indicate better quality of life, whereas higher scores in symptom scales indicate greater symptom burden

Preoperative quality of life showed a weak negative association with postoperative length of stay (*R* = − 0.29, *p* < 0.001), suggesting that patients with higher baseline quality of life tended to have shorter hospitalizations. Results of the multivariable regression analysis showed that the age (*p* = 0.67) and the disease stage (*p* = 0.25) had no significant effect on overall quality of life changes, indicating that the prehabilitation program’s benefits were independent of these factors.

### Anxiety and depression

Similarly to the assessment of quality of life, we assessed the severity of anxiety and depression at the beginning and end of the prehabilitation program. For this purpose, we used the HADS questionnaire.

We have shown that on the day of the first introductory visit, 73 patients (50%) did not exhibit symptoms of anxiety, while 105 patients (72%) did not exhibit symptoms of depression. 35 patients (24%) presented mild anxiety symptoms, 26 (18%) moderate symptoms and 11 patients (7.6%) severe anxiety symptoms. For depression symptoms, the figures were 24 (17%), 10 (6.9%) and 6 (4.1%), respectively. On the day of the first introductory visit the mean score for anxiety was 7.7 (4.2) and for depression 5.3 (4.1).

On the day of admission to the hospital, 87 patients (60%) did not show any symptoms of anxiety, and 111 patients (77%) did not show any symptoms of depression. 33 patients (23%) presented mild anxiety symptoms, 18 (12%) moderate symptoms and 7 patients (4.8%) severe anxiety symptoms. For depression symptoms, the figures were 23 (16%), 9 (6.2%) and 2 (1.4), respectively. For categorical variables, we found no significant differences for the change in anxiety and depression severity during the prehabilitation program, but for continuous variables, we found a significant reduction in the means for anxiety and depression (*p* = 0.003 and *p* = 0.028, respectively). A summary of the results for anxiety and depression, presented both as categorical and continuous variables, is shown in Table 3.

**Table 3:** Comparison of scores for anxiety and depression at the beginning and end of the prehabilitation program

Characteristic	Beginning	End	Difference	*p*-value, *
Anxiety** Mean (SD) Median (IQR)	7.7 (4.2) 7.0 (5.0, 11.0)	6.8 (4.1) 6.0 (4.0, 9.0)	-0.93 (3.36) 0 (-2.5, 1)	0.003 (0.25, small)
Depression** Mean (SD) Median (IQR)	5.3 (4.1) 4.0 (2.0, 8.0)	4.6 (4.0) 4.0 (1.0, 7.0)	-0.69 (3.12) 0 (-2, 1)	0.028 (0.13, small)

*Abbreviations*: *SD* Standard deviation, *IQR* Interquartile range

^*^Effect size (interpretation), ^**^HADS subscale scores range from 0 to 21. Higher scores indicate greater severity of anxiety or depressive symptoms

## Discussion

In our study, we demonstrated a significant improvement in overall quality of life during the prehabilitation period. Our findings confirmed that the preoperative phase was associated with a high prevalence of anxiety and depression (50% and 28%, respectively). Importantly, the mean scores for both anxiety and depression decreased in a statistically and clinically significant but modest over the course of prehabilitation.

Previous investigations have reported a beneficial effect of prehabilitation on quality of life [19–23], on reductions in anxiety and depression [12, 24], or on both outcomes [16, 25–29]. Conversely, some studies failed to show improvements in quality of life [24, 25, 30], reductions in anxiety and depression [31], or either outcome [32, 33]. Notably, many published reports did not assess quality of life or psychological status at all [34–37]. There remains a particular lack of evidence addressing these outcomes in gynecologic oncology, especially among patients with ovarian cancer.

Comparable conclusions regarding quality of life were drawn by Allen et al., who evaluated the effect of prehabilitation in patients receiving neoadjuvant chemotherapy for esophagogastric cancer. Using the QLQ-C30 questionnaire, the authors demonstrated a significant improvement in quality of life in the prehabilitation group. Their psychological component consisted of six medical-coaching sessions delivered in person or by telephone [19].

Similarly, Steinmetz et al. reported that unimodal prehabilitation led to significant preoperative improvements in quality of life among patients awaiting cardiac surgery, despite the absence of a structured psychological intervention [20]. In another trial, a physical-exercise intervention in patients scheduled for lung cancer surgery improved exercise tolerance and produced a significant gain in quality of life [21]. An additional study focusing on patients with stomas demonstrated a marked reduction in anxiety and depression, together with improved quality of life, following participation in a prehabilitation program [9].

Carli et al. [24] reported findings consistent with our results, demonstrating that patients awaiting colorectal surgery exhibited preoperative anxiety and depression levels comparable to those observed in our cohort (mean values of 6 [4] and 4 [3], respectively). In our study, the corresponding mean scores were 7.7 [4.2] for anxiety and 5.3 [4.1] for depression. In the trial by Carli et al., participation in a prehabilitation program resulted in a significant reduction in depressive symptoms (mean change − 0.8 points, *p* < 0.05), whereas anxiety levels remained unchanged.

Furze et al. further demonstrated that prehabilitation prior to cardiac surgery can reduce the incidence of depression in surgical candidates [16].

Conversely, several investigations have not confirmed a beneficial effect of prehabilitation on quality of life [25, 30, 32, 33]. Li et al., in a study assessing trimodal prehabilitation in patients undergoing colorectal cancer surgery, incorporated a psychological component consisting of a 90-minute session with a professional psychologist followed by self-directed implementation of recommendations at home. The median duration of the intervention was 33 days, yet no significant improvement in quality of life was observed during the prehabilitation period [25]. Similarly, Gillis et al. [32] evaluated quality of life and the severity of anxiety and depression at baseline, preoperatively, and postoperatively. Patients received home-based therapies preceded by a one-hour psychological consultation, but no significant preoperative differences emerged between the prehabilitation and control groups. A subsequent study employing a nearly identical psychological protocol likewise failed to demonstrate improvements in self-reported general health status or anxiety and depression [33].

The diagnosis of a malignant tumor is well known to be associated with anxiety, which may progress to depression [38]. Such psychological distress can impair cognitive functioning, diminish quality of life, and reduce overall functional capacity. Without prehabilitation, these factors can lead to inferior perioperative and postoperative outcomes. In our study, as in some previous reports, prehabilitation was associated with improved quality of life and reduced anxiety and depression—key considerations when planning oncologic treatment. Nevertheless, even when prehabilitation does not yield measurable psychological benefits, early identification of patients exhibiting depressive symptoms or heightened anxiety at the time of diagnosis remains critical, as timely intervention can enhance subsequent functioning and treatment outcomes.

In our cohort, we observed either improvements or stability across global health status and individual quality-of-life domains, with no evidence of preoperative deterioration. Notably, despite disease progression, frequent clinical visits, and the stress of a new oncologic diagnosis, neither quality of life nor anxiety and depression scores worsened. Moreover, despite program requirements such as repeated consultations and the purchase of whey protein or vitamin supplements, patients did not report increased financial hardship.

This study has several limitations. First, it was a single-centre, prospective pre–post design without a control group, which prevents definitive attribution of the observed improvements to the prehabilitation program and raises the possibility of natural adaptation or regression to the mean. Second, although statistically significant, the mean increase in global quality-of-life scores (~ 2.5 points) falls below the minimal clinically important difference for the EORTC QLQ-C30, limiting the clinical relevance of the effect. Third, program duration and patient adherence varied widely, and compliance with individual components (exercise, nutrition, psychological support) was not systematically monitored. The duration of the prehabilitation program varied considerably between patients (21.1 ± 16.7 days), which may have influenced the observed outcomes. Patients with longer prehabilitation periods may have had greater opportunity to benefit from the physical, nutritional, and psychological components of the intervention, whereas shorter programs may have limited the potential effect of these measures. Consequently, this variability in program duration could partly contribute to the relatively modest magnitude of the observed improvements. Fourth, no multivariable analyses were performed to adjust for potential confounders such as age, comorbidities, or disease stage. Finally, the single-institution setting may restrict generalizability. Despite these limitations, the study provides valuable preliminary evidence supporting the feasibility and potential psychological benefits of prehabilitation in gynecologic oncology and highlights the need for well-designed randomized controlled trials to confirm the effectiveness of prehabilitation in improving psychological outcomes and quality of life in gynecologic oncology patients. Future research should focus on large, prospective, randomized multicenter trials evaluating the effectiveness of structured prehabilitation programs in patients undergoing gynecologic oncologic surgery. Such studies would help to better determine the true clinical impact of prehabilitation on psychological well-being and perioperative outcomes. Importantly, the psychological dimension of cancer care remains relatively underexplored in gynecologic oncology, despite its potential influence on patients’ quality of life, treatment engagement, and recovery. Further studies examining anxiety, depression, and other aspects of psychological distress during the preoperative period would therefore be highly valuable and could help optimize future prehabilitation strategies.

In conclusion, our study confirms that anxiety and depression are prevalent among patients with gynecologic malignancies. Participation in a multimodal prehabilitation program was associated with improvements in quality of life and reductions in anxiety and depressive symptoms, although the magnitude of these changes was modest. Importantly, we observed no deterioration in psychological well-being or financial status despite the demands of the intervention. Early recognition of depressive or anxiety symptoms in newly diagnosed oncology patients enables prompt initiation of supportive or pharmacologic therapy, thereby improving quality of life and potentially enhancing postoperative outcomes. Oncology centres should prioritize access to professional psychological services, including counseling and psychotherapy, as an integral component of preoperative care.

## Data Availability

The datasets used and/or analyzed during the current study are available from the corresponding author upon reasonable request.
